# The *Jena Eyewitness Research Stimuli (JERS)*: A database of mock theft videos involving two perpetrators, presented in 2D and VR formats with corresponding 2D and 3D lineup images

**DOI:** 10.1371/journal.pone.0295033

**Published:** 2023-12-13

**Authors:** Ulrike Kruse, Stefan R. Schweinberger

**Affiliations:** Department of Psychology, Friedrich Schiller University Jena, Jena, Germany; Kitami Institute of Technology, JAPAN

## Abstract

Empirical investigations into eyewitness identification accuracy typically necessitate the creation of novel stimulus materials, which can be a challenging and time-consuming task. To facilitate this process and promote further research in this domain, we introduce the new *Jena Eyewitness Research Stimuli (JERS)*. They comprise six video sequences depicting a mock theft committed by two different perpetrators, available in both two-dimensional (2D) and 360° format, combined with the corresponding lineup images presented in 2D or three-dimensional (3D) format. Images of one suspect and eight fillers are available for each lineup. We evaluated lineup fairness by using mock eyewitness paradigm and noted a Tredoux’s E of 4.687 for Perpetrator 1 and 5.406 for Perpetrator 2. Moreover, no bias towards the perpetrators was observed in the lineups. We incorporated 360° videos and 3D lineup images to encourage the adoption of innovative data formats in experimental investigations of eyewitness accuracy. In particular, compatibility with Virtual Reality (VR) makes JERS a promising tool for advancing eyewitness research by enabling researchers to construct controlled environments that offer observers an immersive experience. JERS is freely accessible for the use of academic purposes via the Open Science Framework (OSF).

## 1 Introduction

Eyewitness identifications of suspected criminals are often crucial evidence in criminal proceedings. However, it is frequently observed that eyewitnesses exhibit suboptimal accuracy in identification tasks [1–5], and misidentifications of eyewitnesses represent a major factor contributing to wrongful convictions [6]. Since the 1970s, international experimental psychology research has been dedicated to investigating factors that impact person identification, with the aim of mitigating the occurrence of erroneous identifications [2–4, 7].

The development of appropriate stimulus materials for such experiments typically demands a substantial investment of effort, for instance, actors have to be recruited, crime scenes have to be recorded, matching fillers have to be found, lineup images have to be taken and pilot tested. To encourage and facilitate eyewitness research, we here present the *Jena Eyewitness Research Stimuli (JERS)*. This database offers six video sequences of a mock theft involving two different perpetrators in 2D and 360° format, as well as corresponding lineup images in 2D or 3D format. Decades of research in eyewitness and memory studies provide contrasting results on the effects of acute stress on memory [8–11]. The impact of eyewitness stress on lineup accuracy remains unclear [12]. For a more realistic, controlled, and feasible manipulation of stress, JERS includes two 360° videos for usage in VR. According to our current state of knowledge, JERS represents the first publicly available database of stimuli useable for research on eyewitnesses. An overview of all included materials can be found in Table 1.

**Table 1:** Overview of database stimulus material.

	Format	Feature
Video 1	360°	Two perpetrators; victim male
		Two perpetrators; victim female
	2D	Two perpetrators; victim male
		Two perpetrators; victim female
Video 2	360°	Two perpetrators; Perpetrator 1 enters the room first
		Two perpetrators; Perpetrator 2 enters the room first
		Perpetrator 1
		Perpetrator 2
	2D	Two perpetrators; Perpetrator 1 enters the room first
		Two perpetrators; Perpetrator 2 enters the room first
		Perpetrator 1
		Perpetrator 2
Lineup Images	2D	Perpetrator 1: profile shot left and right, half profile shot left and right (45° view), frontal shot
		Eight different fillers regarding Perpetrator 1, each: profile shot left and right, half profile shot left and right (45° view), frontal shot
		Perpetrator 2: profile shot left and right, half profile shot left and right (45° view), frontal shot
		Eight different fillers regarding Perpetrator 2, each: profile shot left and right, half profile shot left and right (45° view), frontal shot
	3D	3D image record of Perpetrator 1
		3D image recordings of eight different fillers for Perpetrator 1
		3D image record of Perpetrator 2
		3D image recordings of eight different fillers for Perpetrator 2
Additional Material	360° movies	“Low stress”: forest scenes
		“High stress”: two scary characters wearing masks appear in a dark basement.

*Note*. In each movie, the camera perspective represents a bystander. Video 1 shows two perpetrators stealing a wallet in a park, with seven other persons present. Video 2 shows two perpetrators stealing a wallet from a backpack in a waiting room, with no other persons present.

## 2 Stimulus development

At the onset of database development, we recruited two dissimilar-looking young adult males to portray the perpetrators in the mock theft scenarios. Both males engage in amateur theatrical performances during their leisure time. Perpetrator 1 was 21 years old at the time of the initial shooting and lineup image production. Perpetrator 2 was 31 years old. The project commenced with the recording of Video 1 (in Sept 2020) and capturing images of the perpetrators and filler individuals for the lineup (Oct 2020—Jan 2023). After a period of two years, we recorded Video 2 (in Sept 2022) featuring the same perpetrators. Subsequent paragraphs provide further elaboration on the development of the stimulus materials.

### 2.1 Videos

All videos were recorded in 360° format using a GoPro™ MAX and edited with the software Adobe™ Premiere Pro 2020 afterwards. This format enables users to look around 360° within the video via a head-mounted display of VR glasses (no further interaction within the video via movement possible). In this way, VR technology creates a simulated 3D experience of reality, and thus is becoming an increasingly important tool for the experimental study of person perception and social interaction [13]. A head-mounted display, placed in front of the user’s eyes, constitutes a dual-screen display accompanied by headphones. The two small screens are positioned individually in front of each eye to allow the presentation of separate computed images that emulate a perception of spatial depth. In addition, head-tracking sensors cause an orientation regarding the user’s current perspective by recording the head movement [14]. Consequently, VR is associated with high immersion and strong perceptions of authenticity regarding the experience [14, 15]. Compared to other media forms, using VR glasses can suggest a strong illusion of a fictional situation to participants that previous experimental psychology concepts could not induce [15]. Accordingly, VR also enables the simulation of risky or ethically problematic situations (e.g. eyewitness experiences) in a safe and controllable laboratory environment [13, 16, 17].

#### 2.1.1 Video 1

Video 1 shows a mock theft scene in a park, captured from the perspective of a bystander who witnessed the crime. The video features the observer on a lawn with seven other individuals (4 females, 3 males; average age = 21.43, *SD* = 3.46, *min* = 16, *max* = 25). All recorded conversations are in German. Perpetrator 1 and Perpetrator 2 appear during the course of the video. Perpetrator 2 initiates a social interaction by approaching a seated individual and requesting a light for his cigarette. The seated individual searches for a lighter in their pocket but is unable to find one and offers an apology. Meanwhile, Perpetrator 1 kneels and covertly picks up an invisible object from the seated individual’s blanket, subsequently stashing the object in his pants pocket. Perpetrator 1 informs Perpetrator 2 that he possesses a lighter at his residence, and the two depart. Subsequently, the target person realizes the theft of their wallet, rises from the ground, vocalizes "Hey, my wallet!" and starts to pursue the two perpetrators, who are now fleeing the scene. The intention of the victim’s loud shouting in the 360° format of the video is to capture the participant’s attention towards the crime and the perpetrators. This sequence was captured twice, once with a male and another with a female victim, to facilitate potential exploration of any influences of victim gender. All actors and actresses involved received a financial allowance of € 20 for the shoot. The two videos have a length of 1:45 min and 1:47 min respectively. The perpetrators are shown in the various sequences and formats from a greater distance (> 3m) and can be seen between 45 and 49 s. Their faces can be seen between 12 and 35 s. For detailed information on the viewing time per actor see S1 Table in S1 File. We considered this time long enough to recognize the perpetrators, as experimental research has shown that even shorter observation times (5 s or more) can be sufficient for correct face recognition [18].

#### 2.1.2 Video 2

To facilitate international implementation and to remove language-specific dialogue as well as to offer videos showing only one perpetrator, four new video sequences were recorded two years later. These videos depict a mock theft occurring in a waiting room. Actors were the same perpetrators as in Video 1. The exhibited items of interior decoration, including a sofa, multiple chairs, and assorted plants, are visually discernible within the recorded footage. At the onset of the video, depending on the video sequence, either Perpetrator 1, Perpetrator 2, or both (age now: 22 and 33, respectively), enter the room. The perpetrators proceed to survey the room before selecting a book from a nearby side table and occupying the sofa to peruse its contents. Upon noticing a backpack leaning against the sofa, their focus shifts from the book to the backpack. The specific sequence of events captured in the video determines which perpetrator is responsible for opening the backpack and retrieving a wallet. The ensuing course of action entails the perpetrators leaving the room. All videos have a length ranging between 1:15–2:29 min. The perpetrators are shown in the various sequences and formats from a closer distance (< 3m) and can be seen between 72 and 143 s. Their faces can be seen between 53 and 96 s. For detailed information on the viewing time per actor see S1 Table in S1 File. Both actors received a financial allowance of € 20 for this second shoot.

### 2.2 Lineup sample

Lineup images were produced for Perpetrator 1 and Perpetrator 2 in 2D and 3D formats. We then photographed eight different German fillers for each lineup, in order to enable an eight-person lineup in which the perpetrator could be present or absent. All images (2 perpetrators, 16 fillers) were shot at the Institute of General Psychology and Cognitive Neuroscience at the Friedrich Schiller University Jena in a standardized form. The fillers were recruited via announcements, flyers and through social networks. All participants received a financial allowance of € 15.

#### 2.2.1 2D images

**Fig 1:**
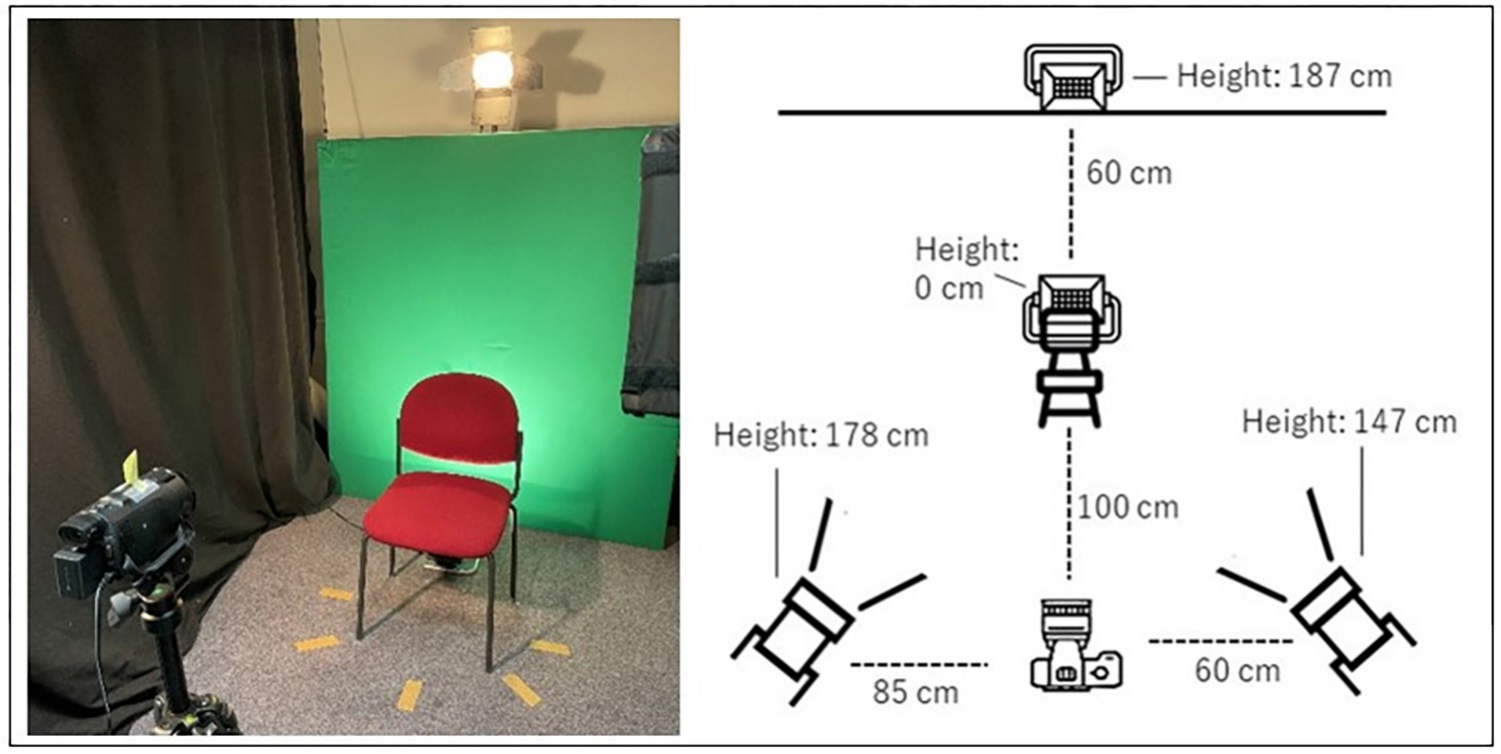
Camera and exposure setup for 2D image creation.

**Fig 2:**
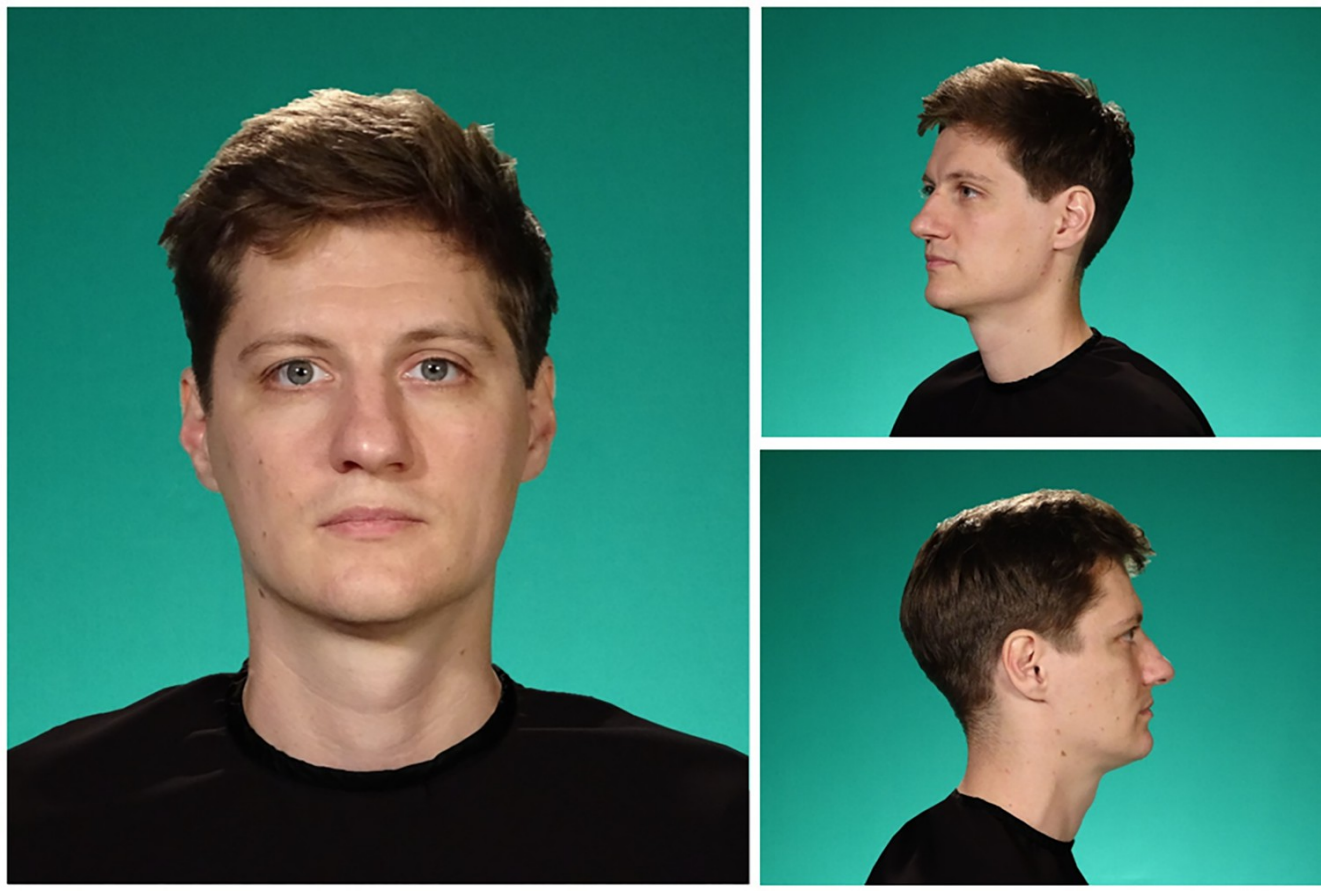
Examples of 2D lineup images. The depicted face shows a filler for Perpetrator 2.

The images were captured using a Canon™ 80D camera, resulting in a 2D format. All participants were seated in front of a green background, wearing a plain black round-neck shirt. Head-and-shoulders-images were obtained of each person from the following perspectives: profile shot left, half profile shot left (45° view), frontal shot, half profile shot right (45° view), profile shot right. The camera and exposure setup are shown in Fig 1. We customized and edited the images using GIMP™ 2.10.32 and Adobe™ Photoshop 23.4.2. Fig 2 shows examples of the 2D lineup images.

#### 2.2.2 3D images

**Fig 3:**
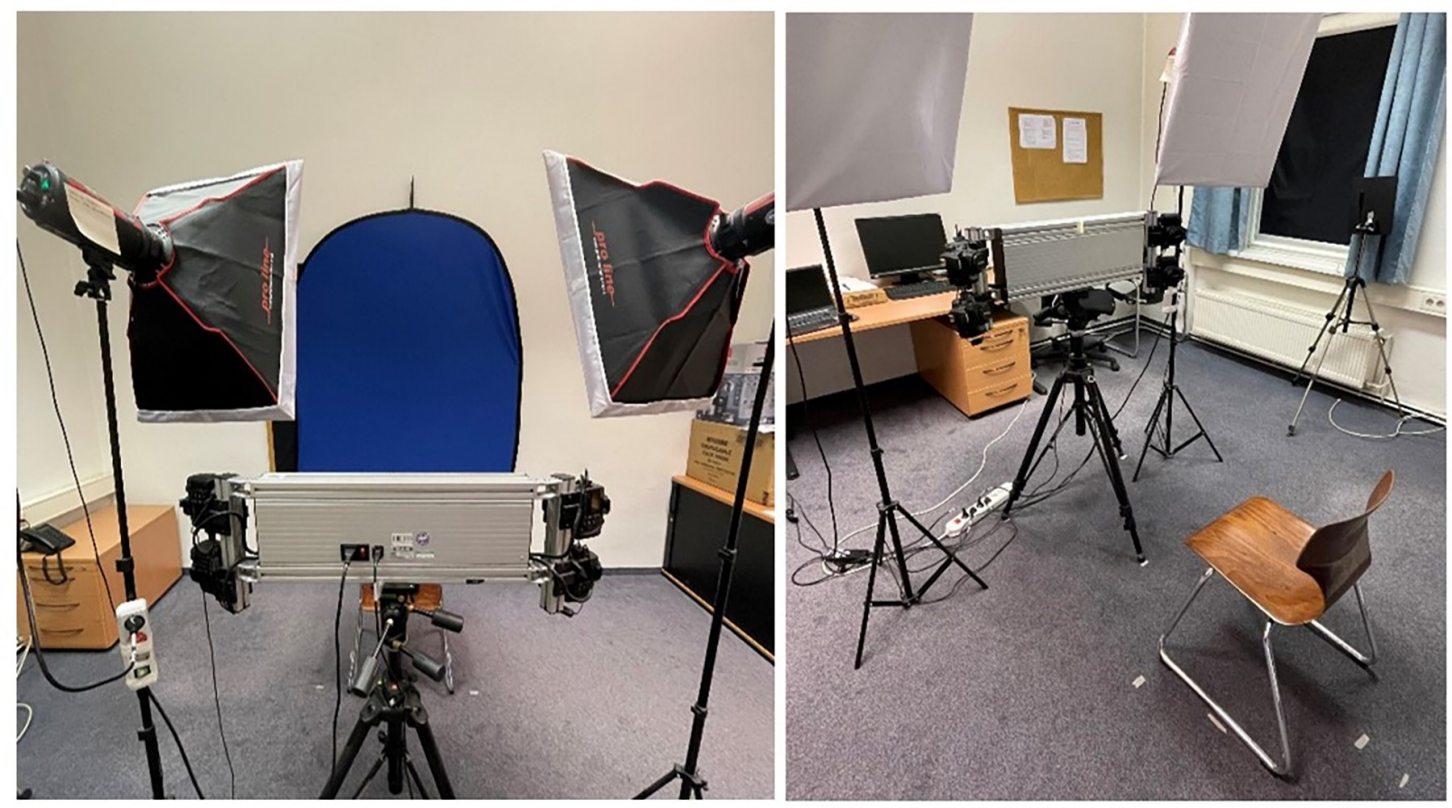
Setup of DI3Dcapture™ system for 3D image creation.

**Fig 4:**
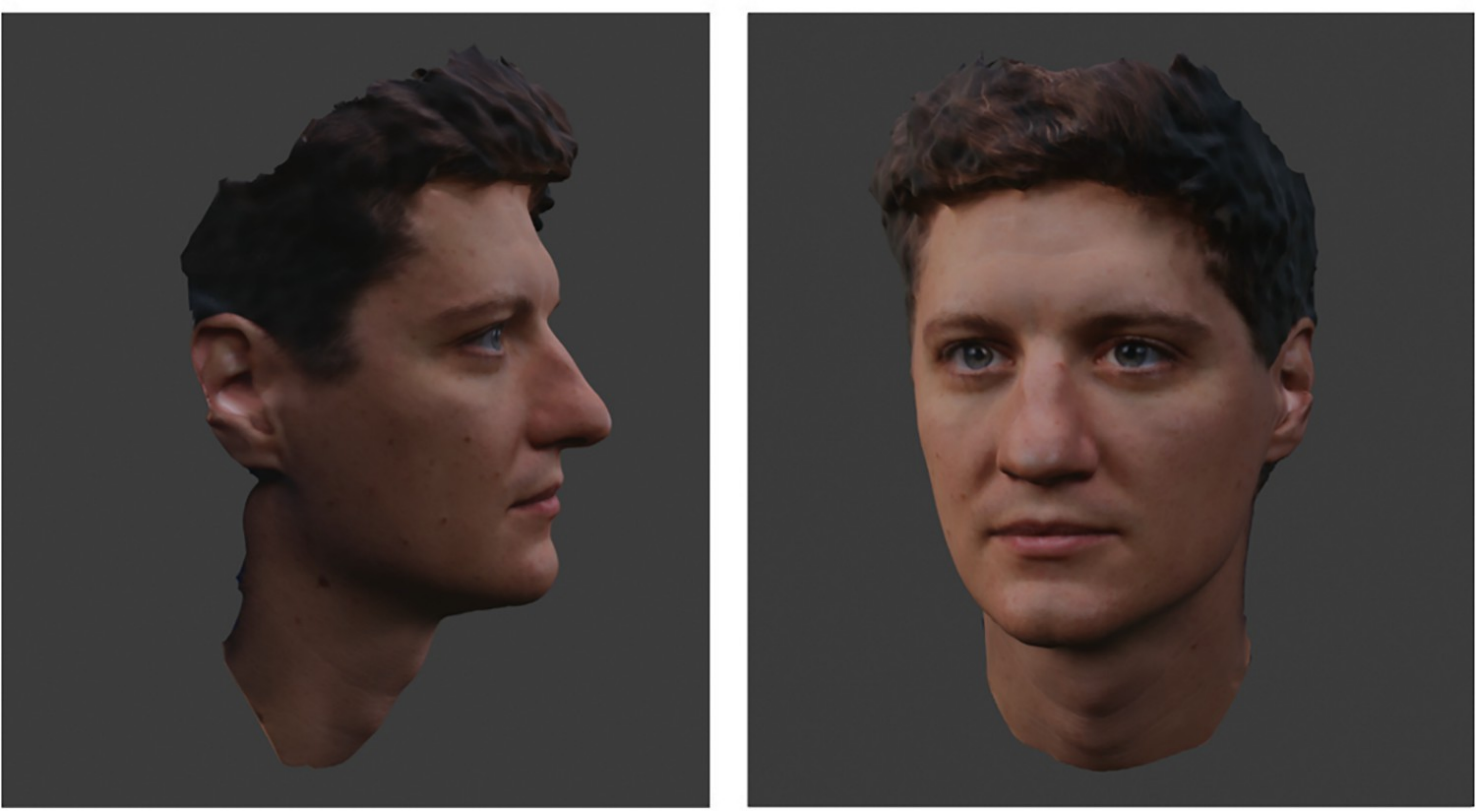
Examples of 3D images (filler for Perpetrator 2;.obj-files possess 3D mobility compared to this figure).

One 3D object file was generated for each of the two perpetrators and all fillers (*n* = 16). Using DI3Dcapture™ (Dimensional Imaging, Glasgow, UK, version 5.2.1), each face was captured by four cameras simultaneously, producing four images for each face that were then interpolated to create a 3D object (.obj). Fig 3 shows the setup of the DI3Dcapture™ system. Example 3D images can be seen in Fig 4. The editing of the 3D objects was performed utilizing the software program Blender 3.2.1.

### 2.3 Testing lineup fairness

The nominal size pertains the number of persons presented in the lineup, while the functional size refers to the number of individuals in the lineup who bear a significant resemblance to the actual suspect [19].

#### 2.3.1 Nominal size

In terms of nominal size, we know that a higher number of fillers produces a lower random risk of identifying a suspect [20]. According to [21], and reflecting common practice in the field, it is advisable to include only one suspect per lineup, with a minimum of five appropriate fillers (see also [22]). Meanwhile, a meta-analysis conducted by Juncu and Fitzgerald [23] has revealed that the inclusion of a greater number of lineup members can enhance the discriminability of suspects. Based on their findings, the authors of the study also recommend a minimum of five fillers to be included in the lineup. Given the significant variations in the recommended minimum number of lineup members across different countries [24], we included lineups with one suspect and eight fillers for the present stimuli. As such, the utilization of these stimuli may be considered appropriate in various countries.

#### 2.3.2 Functional size and lineup bias

Research has shown that distinctive faces are more easily remembered and recognized than non-distinctive, typical faces [25, 26]. To promote fairness and enhance the functional size of a lineup, it is recommended to avoid face images that are highly distinctive [18]. Moreover, it is important to ensure that all fillers in the lineup match any characteristics described by the witness regarding the perpetrator [21, 27]. As such, fairness is a crucial factor to consider in the design of a lineup. In a fair lineup, all face should have similar a priori probabilities of being selected, with none of the faces, including the suspect or any fillers, standing out as being more likely to be chosen, particularly when the target is absent [28–30].

A test of fairness is only available regarding Video 1. Here we used a mock eyewitness paradigm [31, 32]. Accordingly, six independent raters (3 male, 3 female; mean age = 27.5, *SD* = 3.3, *min* = 24, *max* = 32; all born and raised in Germany) were instructed via an anonymous online survey (in Oct 2020) to describe the faces of both perpetrators in their own words on the basis of a sequence of Video 1. They were requested to imagine themselves being called to the police station as a witness and asked to provide a description of the perpetrators. The attributes hair or beard style and its colors, skin color, glasses, age, gender, or special unchangeable distinctive features were given as examples to help verbalizing descriptions. The stimulus used was a 27-second 2D video sequence showing both perpetrators. Raters were allowed to watch the sequence as many times as necessary. All raters’ descriptions were summarized and combined into one modal description for each perpetrator, in which only descriptions that were mentioned by multiple raters were selected. As the majority of raters incorrectly reported an absence of beards for both perpetrators, all raters’ descriptions were included. This approach was taken to avoid a lineup bias caused by this absent-beard description. An overview of the summarized and modal descriptions can be found in S2-S5 Tables in S1 File.

In accordance with the *match to description strategy*, fillers were recruited based on the provided modal descriptions. Subsequently, 130 mock witnesses (not part of the participants described in Section 2.4.1) were instructed to evaluate the respective sets of images (simultaneous format) via an anonymous online survey (Feb—Mar 2023) by choosing the face that best matched the modal description given for each perpetrator. Note that this sample considerably exceed the minimum sample size of 30–50 that is often recommended in this context, which we considered advantageous because choice distributions should become more representative with larger numbers of witnesses (for a similar approach, cf. [33]). The sample was 72.3% female (*n* = 94) and 27.7% male (*n* = 36) with a mean age of 30.6 years (*SD* = 9.4, *min* = 18, *max* = 63). The majority of witnesses were born and raised in Germany (93.8%, *n* = 122), with 3.8% in Austria (*n* = 5) and 2.3% in Switzerland (*n* = 3). In order to determine the effective size of the lineup, Tredoux’s *E* was calculated [27, 34]. This value can vary between 1 and 9 for the present lineups. Here, a value of 1 represents a very unfair and a value of 9 a very fair lineup (ideal performance equivalent to nominal lineup size; cf. [35]). Tredoux’s *E* was 4.687 for the lineup of Perpetrator 1 and 5.406 for the lineup of Perpetrator 2. Detailed data concerning the choice frequencies of the raters is shown in Table 2. This shows that some fillers were selected more often than others. Note that in many real lineup situations, certain fillers are also falsely identified more often than others (e.g., [36]). This situation seems inevitable to some degree, especially when there still exists no commonly accepted metric of facial similarity, and when actual lineup fillers are selected from a limited subset of real people.

**Table 2:** Distribution of mock witness choices in the lineups (*n* = 130).

	Lineup Member								
	1	2	3	4	5	6	7	8	9
Choices in Lineup for Perpetrator 1	34	7	0	9*	18	14	6	0	42
Choices in Lineup for Perpetrator 2	4	0	8	27	14	26	34	17*	0

*Notes*. Perpetrator 1 = Lineup Member 4; Perpetrator 2 = Lineup Member 8; with n perpetrator choices marked with an asterisk.

We examined a possible bias against the perpetrators in each lineup using two measures. First, the proportion of mock witnesses who chose the original perpetrator to match the modal description was tested for significance in relation to the probability that the respective perpetrator was selected by chance using a binomial distribution. In the online survey, 9 witnesses chose Perpetrator 1 and 17 participants chose Perpetrator 2. Given a sample size of *n* = 130, a significance level of 0.05, and a random probability of 0.11, the acceptance range of the binomial distribution falls between the values 8 and 22. Accordingly, the proportion of mock witness choices does not differ significantly from chance in either lineup.

The second measure considers the percentages of the proportions of mock witness choices and chance probability. Ideal performance corresponds with chance, meaning 11% for the present nine-member lineup. The percentages of mock witness choices were 6.9% for Perpetrator 1 (95% CI = 2.6%, 11.3%) and 13.1% for Perpetrator 2 (95% CI = 7.3%, 18.9%). Therefore, there seem to be no bias towards the perpetrators in the lineups. The S1 File contain further details on the characteristics of the mock witnesses, as well as additional data on age and the allocation of lineup members from the mock witness paradigm (Table 2) to the available database (see S6 and S7 Tables in S1 File).

Given the substantial differences between the task of mock witnesses and the task of actual eyewitnesses (c.f., [37, 38]), as well as the potential impact of lineup composition variables such as nominal size and target presence or absence, which can be flexibly manipulated using JERS, we would advise future users of the JERS stimuli to achieve a comprehensive fairness evaluation of any specific lineup version they use based on the lineup data collected (e.g. resultant lineup size, see [37]).

### 2.4 Additional material: Induction of stress

**Fig 5:**
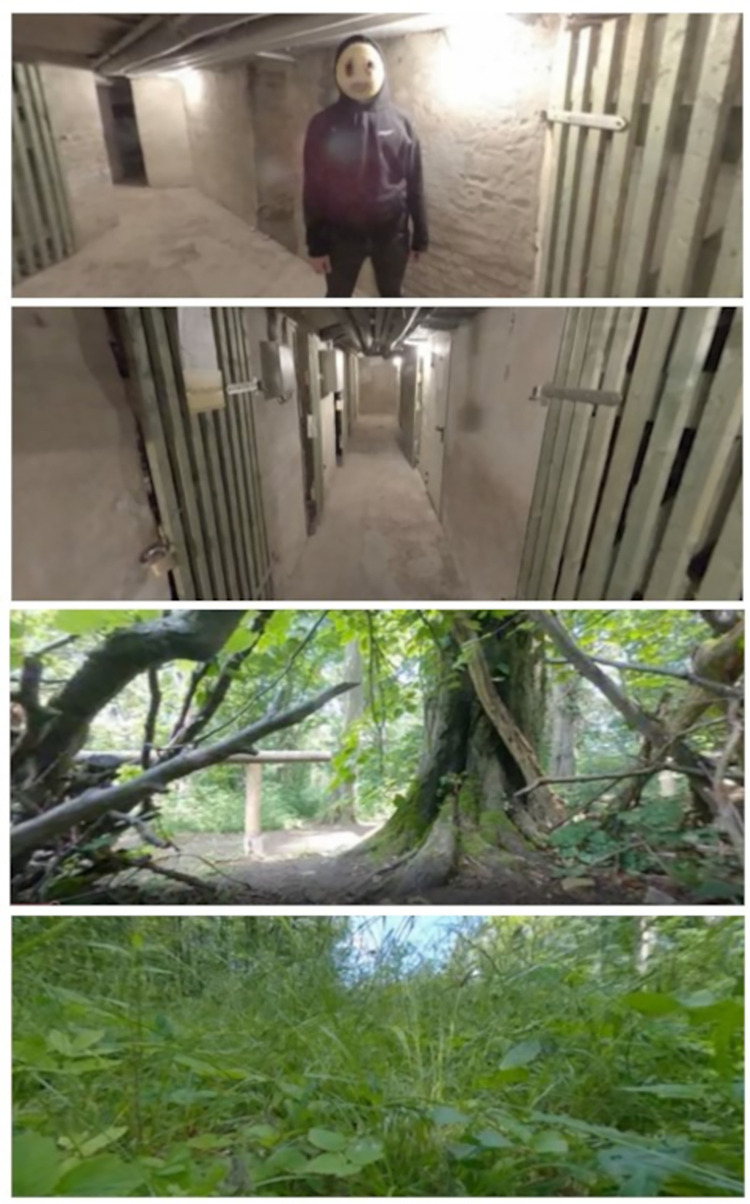
Screenshots of additional material from the “high stress” video with two scenes from the basement with (top image, 1st) and without (2nd) the scary character, and from the “low stress” video (3rd, and bottom image, 4th) with two different forest scenes.

The utilization of a VR technology offers new opportunities for experimental psychological research owing to its ability to engender a heightened sense of authenticity and realism in user experiences [14, 15]. To investigate the impact of stress on the accuracy of eyewitness testimony using a more realistic yet straightforward approach, we recorded two additional videos enabling the manipulation of the participants’ perceived stress level. These include one “high stress” video with two scary characters designed to increase stress levels. It depicts an old, sparsely illuminated basement of an apartment building, accompanied by dark, ominous background music to create a foreboding atmosphere. The video features intermittent periods of darkness. At some point, two individuals wearing white masks appear at various distances from the observer. The final scene of the video shows one of these masked characters running towards the observer. The second “low stress” video shows three forest scenes with the sound of birds in the background, which is intended to have a calming effect on participants. Both videos are in 360° format and viewable with VR glasses. The scary video has a length of 2:48 min and the forest video of 2:49 min. Fig 5 presents various screenshots illustrating the two films.

#### 2.4.1 Validation of stress induction

**Fig 6:**
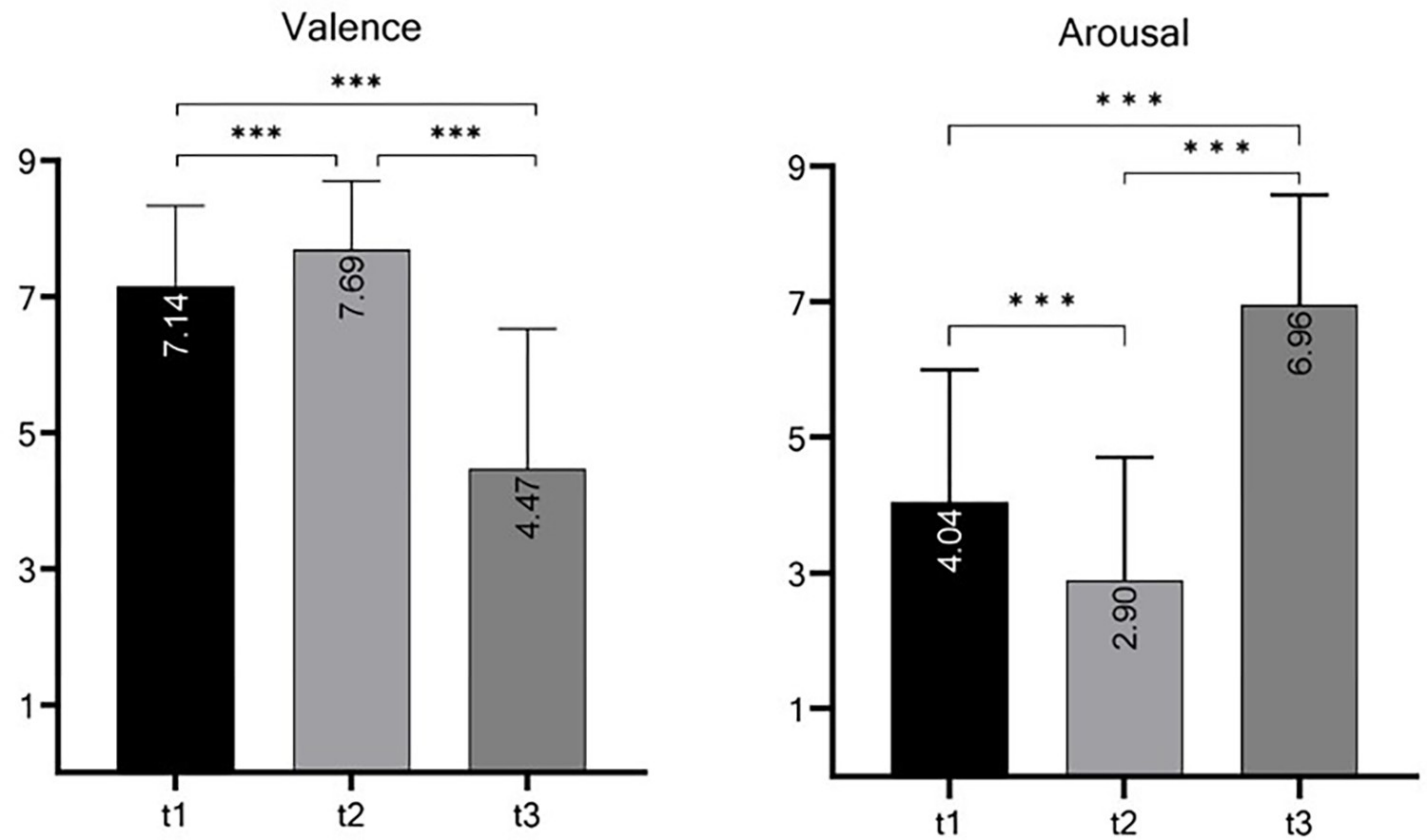
Group differences in *Valence* and *Arousal* prior to viewing the videos (t1), after watching the “low stress” video (t2), and after watching the “high stress” video (t3); *n* = 49, two-tailed paired tested.

**Fig 7:**
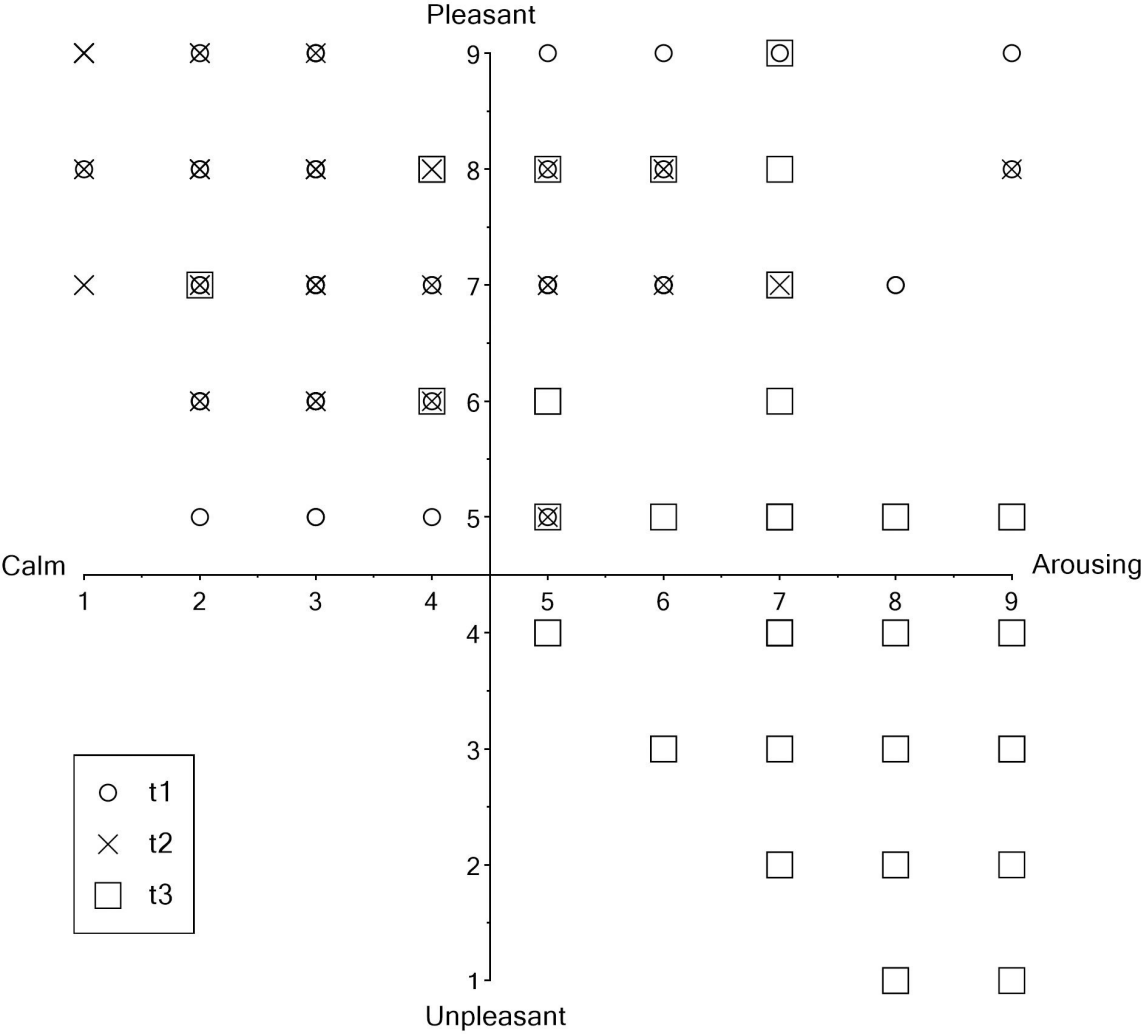
Data distribution (*n* = 49) prior to viewing the videos (t1), after watching the “low stress” video (t2), and after watching the “high stress” video (t3).

To validate the manipulation of stress level induced by the two videos, we used the *Valence* and *Arousal* scales of the Self-Assessment Manikin [39]. For this purpose, 49 participants (10 males, 39 females; mean age = 24.88, *SD* = 10.28, *min* = 18, *max* = 56; additional descriptive data can be found in S8 Table in S1 File) were recruited to watch the “low stress” video followed by the “high stress” video (Nov 2022—Jan 2023). Oculus^TM^ Rift VR glasses and a desktop computer with an NVIDIA GeForce GTX 1060 6GB graphics card were used to present these stimuli. *Valence* and *Arousal* measures were assessed at three different time points: prior to viewing the videos (t1), after watching the “low stress” video (t2), and after watching the “high stress” video (t3). After data collection, the authors did not have access to information that could have identified individual participants. The results displayed in Fig 6 indicate that significant differences in *Valence* and *Arousal* were observed between t1 and t2, t2 and t3, and t1 and t3. Detailed results can be found in S9 Table in S1 File. Furthermore, Fig 7 illustrates that watching the different forest scenes seemed to lead to higher pleasure and calmness, whereas the scary video seemed to induce higher displeasure and arousal. Note that to validate the present stress induction, we only assessed participants’ subjective perception of their emotions. Given the multifaceted nature of stress (in terms of subjective, behavioral, and physiological responses), further validation of stress induction by these videos might be desirable and could be collected when using these videos in the future. These could include assessing physiological stress indicators (e.g., heart rate, blood pressure, skin conductance or cortisol levels), or behavioral measures (e.g., quantification of avoidance behavior via 3D motion tracking).

### 2.5 Ethics statement and research on human participants

The research reported in this article has been approved by the Ethics Committee of the Faculty for Social and Behavioral Sciences of Friedrich Schiller University (Reg.-Nr. FSV 20/035). All participants provided written informed consent to their participation; moreover, all participants appearing in the images or videos of the stimulus database provided written informed consent to the non-commercial use of these stimuli in scientific research, and to their inclusion and publication in the context of this database. The individual in this manuscript has given written informed consent (as outlined in PLOS consent form) to publish these case details.

## 3 Potential use of the stimuli

There is high and increasing demand for, and use of, lineup stimuli in psychological eyewitness research. For instance, a Web of Science search of scientific articles using the keyword ’lineup’ (conduced on 22 Mai 2023) returned more than 1,200 papers published since January 1^st^, 2000, as compared with 360 papers published in the years 1975–1999. However, as the creation and validation of appropriate stimuli take substantial time and effort, we hope that by providing JERS to the research community, we will facilitate further eyewitness research and encourage the use of new data formats in eyewitness accuracy studies. Especially the utilization of VR videos and 3D lineup images appears to be inadequately represented despite its potential, which may be attributed to the perceived high levels of expertise, equipment and resources required for generating such stimuli. JERS is freely accessible for the use of academic purposes via the Open Science Framework (OSF; https://osf.io/c29h6/; DOI: 10.17605/OSF.IO/C29H6).

The importance of conducting eyewitness research stems from the recognition that eyewitness reports may be vulnerable to inaccuracies, despite their considerable influence in criminal justice proceedings [2–4]. Consequently, it is crucial to comprehend the precision of eyewitness reports and facilitate their interpretation to guarantee equitable verdicts. JERS provides a diverse range of possibilities for conducting eyewitness accuracy research, encompassing both laboratory and online settings. The availability of different video variants allows the investigation of various factors in addition to lineup perpetrator identification, such as the impact of the victim’s gender, the presence of single or multiple perpetrators (e.g., see [40], or the distinction between 2D and 360° videos shown under conventional versus VR conditions (e.g., [41, 42]. Video 1 with long-distance shots of the perpetrators represents a realistic scenario of observing a theft from the perspective of an unrelated witness sitting in a park. Also, to allow for more specific investigations that require a longer time for facial encoding (e.g., recognition of facial expressions, emotions, or specific facial features), Video 2 shows extended illustrations of the perpetrators from a short distance. Please note when using the videos that these theft scenes are not necessarily representative of other crimes, especially those involving more violence or weapons. Also noteworthy is the circumstance that Video 2 was recorded two years after the lineup images were captured, so differences in the appearance of the perpetrators are visible. Accordingly, this enables the investigation of eyewitness accuracy despite changes in appearance, for instance due to different hair lengths. A real-life example could be that an individual who has already committed a crime, and of whom the police already have photographs, becomes a new suspect, a situation in which witnesses may be shown the older images.

Furthermore, while we appreciate that some researchers who wish to use the JERS stimuli to investigate effects or stress or emotions on eyewitness memory might use their own or customized material (e.g., for a large stimulus database, see [43], the integration of additional materials to induce stress into JERS has potential advantages of compatibility, including seamless integration into VR presentation settings.

In fact, VR represents a promising tool for eyewitness research, as it enables researchers to create controlled environments that provide observers with an immersive experience [13–15]. The use of VR facilitates the creation of realistic situations, allowing participants to feel as eyewitnesses to a crime or event.

In addition, we emphasize that our present inclusion of 3D lineup images opens a promising avenue for research, and we hope that researchers using the JERS will find this a useful resource which warrants further investigation. Law enforcement agencies are increasingly turning to various forms of 3D image usage, in addition to the commonly employed 2D photographs of suspects [44]. For instance, in Tokyo, the Japanese police had begun using 3D images when photographing arrested individuals already in 2016 [45], while in Germany, researchers have been working on the GES-3D project (2012–2015) to develop a multi-biometric system that utilizes 3D facial images for the identification of crime suspects, even from low-quality photo or video data [46]. Consequently, more widespread use of 3D image recordings as lineup images appears to be only a matter of time. From an experimental perspective, 3D images of faces permit researchers to display lineup faces across a large range of viewpoints as required by their specific research question. From an applied perspective, the potential for additional information available in 3D images [47] to enhance eyewitness identification accuracy (compared with 2D images) will be of primary interest.

## Supporting information

S1 Checklist STROBE statement—checklist of items that should be included in reports of observational studies. (PDF)

S1 File (PDF)
