# Supplementary material for: The Jena Eyewitness Research Stimuli (JERS): A database of mock theft videos involving two perpetrators, presented in 2D and VR formats with corresponding 2D and 3D lineup images
Source: PLoS One. 2023 Dec 13;18(12):e0295033. doi: 10.1371/journal.pone.0295033 (PMC10718457; doi:10.1371/journal.pone.0295033)
Supplement: S1 Checklist — (PDF) [file pone.0295033.s001.pdf]

STROBE Statement—checklist of items that should be included in reports of observational studies

	Item No.	Recommendation	Page No.	Relevant text from manuscript
**Title and abstract**	1	(*a*) Indicate the study's design with a commonly used term in the title or the abstract		*Title*
		(*b*) Provide in the abstract an informative and balanced summary of what was done and what was found		*Abstract*
**Introduction**				
Background/rationale	2	Explain the scientific background and rationale for the investigation being reported	1	Covered in *Introduction*
Objectives	3	State specific objectives, including any prespecified hypotheses	1<br>7-10<br>10	*Introduction*<br>*2.3 Testing lineup fairness*<br>*2.4 Additional Material: Induction of stress*
**Methods**				
Study design	4	Present key elements of study design early in the paper	1<br>7-10<br>11	Described in *Introduction* and in-depth in *2.3.2 Functional size and lineup bias* and *2.4.1 Validation of stress induction*
Setting	5	Describe the setting, locations, and relevant dates, including periods of recruitment, exposure, follow-up, and data collection	3-11	*2 Stimulus development*
Participants	6	(*a*) *Cohort study*—Give the eligibility criteria, and the sources and methods of selection of participants. Describe methods of follow-up<br>*Case-control study*—Give the eligibility criteria, and the sources and methods of case ascertainment and control selection. Give the rationale for the choice of cases and controls<br>*Cross-sectional study*—Give the eligibility criteria, and the sources and methods of selection of participants	7-10<br>11	*2.3.2 Functional size and lineup bias*<br>*2.4.1 Validation of stress induction*
		(*b*) *Cohort study*—For matched studies, give matching criteria and number of exposed and unexposed		NA

		Case-control study—For matched studies, give matching criteria and the number of controls per case		
Variables	7	Clearly define all outcomes, exposures, predictors, potential confounders, and effect modifiers. Give diagnostic criteria, if applicable	3-11	*2 Stimulus development*
Data sources/ measurement	8*	For each variable of interest, give sources of data and details of methods of assessment (measurement). Describe comparability of assessment methods if there is more than one group	3-11	*2 Stimulus development*
Bias	9	Describe any efforts to address potential sources of bias	7-10<br>11	*2.3.2 Functional size and lineup bias*<br>*2.4.1 Validation of stress induction*
Study size	10	Explain how the study size was arrived at	7-10	*2.3.2 Functional size and lineup bias*

Quantitative variables	11	Explain how quantitative variables were handled in the analyses. If applicable, describe which groupings were chosen and why	7-10<br>11	*2.3.2 Functional size and lineup bias*<br>*2.4.1 Validation of stress induction*
Statistical methods	12	(*a*) Describe all statistical methods, including those used to control for confounding	7-10<br>11	*2.3.2 Functional size and lineup bias*<br>*2.4.1 Validation of stress induction*
		(*b*) Describe any methods used to examine subgroups and interactions		NA
		(*c*) Explain how missing data were addressed		NA
		(*d*) *Cohort study*—If applicable, explain how loss to follow-up was addressed<br>*Case-control study*—If applicable, explain how matching of cases and controls was addressed<br>*Cross-sectional study*—If applicable, describe analytical methods taking account of sampling strategy		NA
		(*e*) Describe any sensitivity analyses		NA
**Results**				
Participants	13*	(a) Report numbers of individuals at each stage of study—eg numbers potentially eligible, examined for eligibility, confirmed eligible, included in the study, completing follow-up, and analysed	7-10<br>11	*2.3.2 Functional size and lineup bias*<br>*2.4.1 Validation of stress induction*
		(b) Give reasons for non-participation at each stage	8	All born and raised in German speaking countries
		(c) Consider use of a flow diagram		NA

Descriptive data	14*	(a) Give characteristics of study participants (eg demographic, clinical, social) and information on exposures and potential confounders	3<br>7-10<br>11	*2 Stimulus development*<br>*2.3.2 Functional size and lineup bias*<br>*2.4.1 Validation of stress induction + Supplemental Material*
		(b) Indicate number of participants with missing data for each variable of interest		NA/*Supplemental Material*
		(c) *Cohort study*—Summarise follow-up time (eg, average and total amount)		NA
Outcome data	15*	*Cohort study*—Report numbers of outcome events or summary measures over time		NA
		Case-control study—Report numbers in each exposure category, or summary measures of exposure		NA
		Cross-sectional study—Report numbers of outcome events or summary measures	7-10<br>11	*2.3.2 Functional size and lineup bias*<br>*2.4.1 Validation of stress induction*
Main results	16	(*a*) Give unadjusted estimates and, if applicable, confounder-adjusted estimates and their precision (eg, 95% confidence interval). Make clear which confounders were adjusted for and why they were included	7-10	*2.3.2 Functional size and lineup bias*
		(*b*) Report category boundaries when continuous variables were categorized		NA
		(*c*) If relevant, consider translating estimates of relative risk into absolute risk for a meaningful time period		NA

Other analyses	17	Report other analyses done—eg analyses of subgroups and interactions, and sensitivity analyses		NA
**Discussion**				
Key results	18	Summarise key results with reference to study objectives	7-10<br>11	*2.3.2 Functional size and lineup bias*<br>*2.4.1 Validation of stress induction*
Limitations	19	Discuss limitations of the study, taking into account sources of potential bias or imprecision. Discuss both direction and magnitude of any potential bias	11	*2.4.1 Validation of stress induction*
Interpretation	20	Give a cautious overall interpretation of results considering objectives, limitations, multiplicity of analyses, results from similar studies, and other relevant evidence	7-10<br>11	*2.3.2 Functional size and lineup bias*<br>*2.4.1 Validation of stress induction*
Generalisability	21	Discuss the generalisability (external validity) of the study results	11	*2.4.1 Validation of stress induction*
**Other information**				
Funding	22	Give the source of funding and the role of the funders for the present study and, if applicable, for the original study on which the present article is based		Given in submission document

*Give information separately for cases and controls in case-control studies and, if applicable, for exposed and unexposed groups in cohort and cross-sectional studies.

Note: An Explanation and Elaboration article discusses each checklist item and gives methodological background and published examples of transparent reporting. The STROBE checklist is best used in conjunction with this article (freely available on the Web sites of PLoS Medicine at http://www.plosmedicine.org/, Annals of Internal Medicine at http://www.annals.org/, and Epidemiology at http://www.epidem.com/). Information on the STROBE Initiative is available at www.strobe-statement.org.
