# Supplementary material for: The Jena Eyewitness Research Stimuli (JERS): A database of mock theft videos involving two perpetrators, presented in 2D and VR formats with corresponding 2D and 3D lineup images
Source: PLoS One. 2023 Dec 13;18(12):e0295033. doi: 10.1371/journal.pone.0295033 (PMC10718457; doi:10.1371/journal.pone.0295033)
Supplement: S1 File — (PDF) [file pone.0295033.s002.pdf]

Supporting Information

Table S1

Viewing time per actor (in seconds).

	Format	Feature		Distant Shots (> 3 m distance)	Close-Ups (< 3m distance)	Frontal Face View	Other Face View	Non-Facial View
Video 1	360°	Two Ps; V male	P1	46	0	5	7	34
			P2	48	0	5	13	30
		Two Ps; V female	P1	46	0	9	17	20
			P2	49	0	5	30	14
	2D	Two Ps; V male	P1	46	0	5	7	34
			P2	48	0	5	13	30
		Two Ps; V female	P1	46	0	9	17	20
			P2	45	0	5	26	14
Video 2	360°	Two Ps; P1 enters the room first	P1	11	77	16	52	20
			P2	17	69	23	54	9
		Two Ps; P2 enters the room first	P1	16	62	18	48	12
			P2	9	69	13	55	10
		P1		17	126	21	75	47
		P2		13	59	12	41	19
	2D	Two Ps; P1 enters the room first	P1	11	77	16	52	20
			P2	17	69	23	54	9
		Two Ps; P2 enters the room first	P1	16	62	18	48	12
			P2	9	69	13	55	10
		P1		17	126	21	75	47
		P2		13	59	12	41	19

Notes. P = Perpetrator, V = Victim

Table S2

Summarized person descriptions regarding Perpetrator 1.

**Attribute**	**Descriptions mentioned (*n*)**
Sex	Male (6)
Age	Mid 20s (3)
	Early 20s (1)
	20-25 years (1)
	20-30 years (1)
Skin Color	White (6), pale (2)
Hair Color	Brown (2)
	Dark brown - black (1)
	Dark (1)
	Black (1)
Hairstyle	Straight (2)
	Long (2)
	Chin-length (1)
	Chin-shoulder-length (2)
	Shoulder-length (1)
Beard	No beard (4)
	Short dark chin beard (1)
Glasses/Jewellery	No glasses (1)
Description of facial features	Roundish face (1)
	Pointy facial features (1)
	Feminine face shape (1)

Note. Description counts can include multiple descriptions per rater and attribute, where applicable.

Table S3

Summarized person descriptions regarding Perpetrator 2.

**Attribute**	**Descriptions mentioned (*n*)**
Sex	Male (6)
Age	Late 20s (1)<br>Mid 20s (2)<br>Early 20s (1)<br>Between 20 and 30 (1)<br>Between 25 and 30 (1)
Skin Color	White (6), little tanned (1)
Hair Color	Blonde (3), blonde/brown (1), Dark blonde (1)
Hairstyle	Short (4)<br>Approx. 4-5cm long (2)<br>Curly (2)<br>Medium length (1)<br>"Parted on the left side, hair hang down into the face, ears free, little sideburns" (1)
Beard	No beard (2)<br>Small upper lip beard (1)<br>Shaved - max. stubbly beard (1)<br>Beard below chin (1)
Glasses/Jewellery	No glasses, No face jewellery (1)
Description of facial features	Nose relatively large (1),<br>Straight nose (1),<br>Elongated nose (1)<br>Low-set eyes (1)<br>Distinctive facial bones (1)<br>Soft facial features (1)<br>Inconspicuous face (1)<br>Short chin (1)

Note. Description counts can include multiple descriptions per rater and attribute, where applicable.

Table S4

Modal description Perpetrator 1.

**Attribute**	**Description**
Gender	Male
Age	Between 20 and 30 years old, rather early-mid 20s.
Skin Color	White, rather pale
Hair Color	Black-brown
Hairstyle	Straight, chin-shoulder length
Beard	No beard - short dark chin beard

Table S5

Modal description Perpetrator 2.

**Attribute**	**Description**
Gender	Male
Age	Between 20 and 30 years old, rather mid-late 20s
Skin Color	White
Hair Color	Dark blonde-blonde
Hairstyle	Short, curly hair, approx. 4-5 cm long
Beard	No beard - stubbly beard/small upper lip beard/beard below chin

Table S6

Additional lineup data.

Database label	Age	Lineup member in mock witness paradigm
Perpetrator 1	21	4
P1_Filler 1	22	1
P1_Filler 2	30	3
P1_Filler 3	25	6
P1_Filler 4	26	7
P1_Filler 5	24	5
P1_Filler 6	25	8
P1_Filler 7	22	2
P1_Filler 8	22	9
Perpetrator 2	31	8
P2_Filler 1	25	3
P2_Filler 2	33	2
P2_Filler 3	27	9
P2_Filler 4	26	5
P2_Filler 5	27	4
P2_Filler 6	23	1
P2_Filler 7	21	7
P2_Filler 8	30	6

Table S7

Descriptive statistic of mock witness sample size variables in absolute (n) and relative (%) frequencies (n =130).

	n (%)
Occupation (*n* = 128)	
Student	62 (48.4)
Employed	50 (39.1)
Official	5 (3.9)
Self-employed	3 (2.3)
Unemployed	3 (2.3)
Apprentice	2 (1.6)
Housewife/Houseman	1 (0.8)
Pupil	1 (0.8)
Retired	1 (0.8)
Student's degree subject (*n* = 62)	
Psychology	18 (29.0)
Business Administration	14 (22.6)
Media Studies	7 (11.3)
Economics	6 (9.7)
Economic Psychology	3 (4.8)
Management	2 (3.2)
Medicine	2 (3.2)
Anglistics	1 (1.6)
Animation & Vfx	1 (1.6)
Behavioural Sciences	1 (1.6)
E-Commerce	1 (1.6)
Educational Sciences	1 (1.6)
Linguistics	1 (1.6)
Plural Economics	1 (1.6)
Politics	1 (1.6)
Psychology of Consumers and Market Research	1 (1.6)
Public Communication	1 (1.6)
Color blindness (*n* = 130)	0 (0.0)

Table S8

Descriptive statistic of sample size variables in absolute (n) and relative (%) frequencies regarding validation of stress induction

	n (%)
Occupation (*n* = 49)	
Student	42 (85.7)
Employed	6 (12.2)
Unemployed	1 (2.0)
Student's degree subject (*n* = 42)	
Psychology	31 (73.8)
Industrial Engineering	3 (7.1)
Geological Sciences	2 (4.8)
Sociology	2 (4.8)
Communication Sciences	1 (2.4)
Educational Sciences	1 (2.4)
History of Art	1 (2.4)
Philosophy	1 (2.4)
Visual impairment (*n* = 49)	16 (32.7)
Normal visual acuity is achieved with visual aids (*n* = 49)	16 (32.7)
Visual aids are worn during experiments (*n* = 49)	16 (32.7)
Color blindness (*n* = 49)	0 (0.0)

Table S9

Difference calculations (paired) between t1 (prior to video presentation), t2 (after having perceived the "low stress" forest video) and t3 (after having perceived the "high stress" scary video) regarding Valence and Arousal (n = 49). Larger scores indicate more arousal and more positive valence, respectively.

	t1		t2					
	M	*SD*	*M*	*SD*	*t*(*df*)	*p*	95% CI	*Cohen's d*
Arousal	4.04	1.96	2.90	1.81	4.753 (48)	.000***	[.659, 1.626]	.679
Valence	7.14	1.19	7.69	1.00	-3.852(48)	.000***	[-.839, -.263]	-.550
	t2		t3					
	M	*SD*	*M*	*SD*	*t*(*df*)	*p*	95% CI	*Cohen's d*
Arousal	2.90	1.81	6.96	1.62	-11.550(48)	.000***	[-4.768, -3.354]	-1.650
Valence	7.69	1.00	4.47	2.05	9.543(48)	.000***	[2.545, 3.904]	1.363
	t1		t3					
	M	*SD*	*M*	*SD*	*t*(*df*)	*p*	95% CI	*Cohen's d*
Arousal	4.04	1.96	6.96	1.62	-7.795(48)	.000***	[-3.671, -2.166]	-1.114
Valence	7.14	1.19	4.47	2.05	9.105(48)	.000***	[2.083, 3.264]	1.301

Notes. ***$p \leq .001$. Two-tailed tested. CI = Confidence Interval.
